# Deletion of the Scl +19 enhancer increases the blood stem cell compartment without affecting the formation of mature blood lineages

**DOI:** 10.1016/j.exphem.2012.02.006

**Published:** 2012-07

**Authors:** Dominik Spensberger, Ekaterini Kotsopoulou, Rita Ferreira, Cyril Broccardo, Linda M. Scott, Nasios Fourouclas, Katrin Ottersbach, Anthony R. Green, Berthold Göttgens

**Affiliations:** Department of Haematology, Cambridge Institute for Medical Research, University of Cambridge, Cambridge, UK

## Abstract

The stem cell leukemia (*Scl*)*/Tal1* gene is essential for normal blood and endothelial development, and is expressed in hematopoietic stem cells (HSCs), progenitors, erythroid, megakaryocytic, and mast cells. The Scl +19 enhancer is active in HSCs and progenitor cells, megakaryocytes, and mast cells, but not mature erythroid cells. Here we demonstrate that in vivo deletion of the Scl +19 enhancer (*Scl*^*Δ19/Δ19*^) results in viable mice with normal Scl expression in mature hematopoietic lineages. By contrast, Scl expression is reduced in the stem/progenitor compartment and flow cytometry analysis revealed that the HSC and megakaryocyte-erythroid progenitor populations are enlarged in *Scl*^*Δ19/Δ19*^ mice. The increase in HSC numbers contributed to enhanced expansion in bone marrow transplantation assays, but did not affect multilineage repopulation or stress responses. These results affirm that the Scl +19 enhancer plays a key role in the development of hematopoietic stem/progenitor cells, but is not necessary for mature hematopoietic lineages. Moreover, active histone marks across the Scl locus were significantly reduced in *Scl*^*Δ19/Δ19*^ fetal liver cells without major changes in steady-state messenger RNA levels, suggesting post-transcriptional compensation for loss of a regulatory element, a result that might be widely relevant given the frequent observation of mild phenotypes after deletion of regulatory elements.

The stem cell leukemia (*Scl*) gene, also known as *Tal1*, encodes a basic helix-loop-helix transcription factor that functions as a critical regulator of both hematopoietic and endothelial development [1]. SCL was first identified by virtue of its ectopic expression as a target of t(1;14) chromosomal translocations in T-cell acute lymphoblastic leukemia [2]. Overexpression of SCL is now recognized as one of the most common molecular abnormalities found in human T-cell acute lymphoblastic leukemia [3].

Scl is an essential regulator of the hematopoietic hierarchy at several levels. Within the hematopoietic lineage, Scl is expressed in hematopoietic stem cells (HSCs), progenitor cells, and in erythroid, megakaryocytic, and mast cells [4–6]. *Scl* null ES cells fail to differentiate in vitro and do not contribute in vivo to hematopoiesis in chimeric mice [6,7]. In addition, knockout of the *Scl* gene is embryonic lethal at E9.5, due to complete absence of hematopoiesis and major vascular defects [7–9]. More recently, the use of a conditional knockout has demonstrated that Scl is essential for the genesis, but not the maintenance, of HSCs [10,11]. Mice in which *Scl* was deleted in adulthood exhibited mild defects in erythropoiesis and megakaryopoiesis [11] and increased Lin^−^cKit^+^Sca^+^ stem-cell enriched population [12]. Short-term HSC (ST-HSC) function seems to be defective in Scl deleted cells because these cells fail to generate colony-forming unit (CFU)-S12 colonies in the spleen [10] and show reduced short-term repopulating ability [12]. Interestingly, long-term HSC (LT-HSC) function was not compromised [11] or mildly compromised [12] when the *Scl* deletion occurred post-transplantation. However, if the deletion occurred before transplantation, then a reduction in repopulating ability of the deleted cells was observed, which was not due to homing defects [12]. This defect in repopulating ability was already observed in heterozygous Scl deleted cells, indicating that haploinsufficiency is enough to affect the repopulation capacity of these cells [12]. Reduction of Scl expression using short hairpin RNA lentivirus in both human and mouse stem-cell enriched populations also affects the short and long-term repopulating ability of these cells [13].

A systematic survey of the promoters and chromatin structure of the murine *Scl* gene has identified several regulatory elements, functionally validated in reporter assays [14–18]. Further analysis of reporter constructs in transgenic mice identified a panel of spatially distinct enhancers, each of which directs *Scl* expression to a subdomain of the normal *Scl* expression pattern [14,16–18]. In particular, the Scl +19 enhancer, also known as the Scl +18/19 enhancer from its location 19 kb downstream of the Scl promoter, was shown to drive expression of *Scl* in long-term repopulating HSCs and hematopoietic progenitors, but not in mature cells [17,19]. Furthermore, expression of the *Scl* complementary DNA under the control of the Scl +19 enhancer rescued the formation of early hematopoietic progenitors and yolk sac angiogenesis in *Scl*^−/−^ embryos, but failed to rescue erythropoiesis and embryos still died at E9.5 [19]. These results indicate that the Scl +19 enhancer plays an important role in progenitors but is not sufficient to support erythroid maturation.

Transgenic mouse reporter assays are a useful tool to identify new regulatory elements; however, such approaches are unable to define nonredundant/essential roles of these elements in the context of the entire gene locus. In the case of the Scl gene, three hematopoietic enhancers have been described that, in combination, are responsible for the hematopoietic expression pattern of Scl [14,16–18]. These enhancers have evolved from common ancestral enhancers [20] and may have maintained a certain degree of redundancy.

To clarify the function of the Scl +19 enhancer within the context of the endogenous locus, we describe here the generation and analysis of mice lacking both copies of the Scl +19 enhancer (*Scl*^*Δ19/Δ19*^). *Scl*^*Δ19/Δ19*^ mice were viable but their HSC and megakaryocyte-erythroid progenitor compartments were expanded. Analysis of Scl expression as well as chromatin modification status in wild-type (WT) and mutant cells suggested that post-transcriptional compensatory mechanisms contribute to the mild phenotype in addition to redundant regulatory elements within the locus.

## Materials and methods

### Mice, genotyping, and breeding

Mice with a +18/19 targeted stem cell enhancer (*Scl*^*Δ19/Δ19*^) were generated as described [16]. Mice and tissues were routinely genotyped by polymerase chain reaction (PCR) using the following primers: WT allele, 5′-CACCTGTCCTGGGGCTAAATT-3′ and 5′-GTTTTTGACTCCCAGATGTTGAA-3′; +18/19 enhancer region deletion allele (Δ19), 5′-CTTCTATCCATCTACAGG-3′ and 5′-CACTGAATCATGCTCGTGTGG-3′. Animals were maintained in the Cambridge Central Biomedical Services in accordance with institutional guidelines.

### Peripheral blood analysis, cell staining, and flow cytometry

A sample (50 μL) of freshly isolated peripheral blood from the tail vein was collected and blood parameters were measured using an ABC Vet fully automated analyzer (ABX Hematologie, Montpellier, France). For hematopoietic precursor isolation, mature bone marrow (BM) cells were depleted with a lineage depletion column (Miltenyi Biotec, GmBH, Bergisch Gladbach, Germany). For identification of common myeloid progenitors, granulocyte-macrophage progenitors, and megakaryocytic erythroid progenitors, cells were incubated with allophycocyanin anti-c-Kit (2B8; Pharmingen), phycoerythrin anti-FcγRII/III (2.4G2; Pharmingen BD Biosciences, Oxford, UK), and fluorescein isothiocyanate (FITC) anti-CD34 (RAM34; Pharmingen). Common lymphoid progenitors cells among Lin^−^ cells were enumerated after staining with allophycocyanin c-Kit, Pacific Blue Sca-1 (E13-161.7; Pharmingen), and biotin-conjugated interleukin-7 (B12-1; Pharmingen), followed by PerCP-Cy5.5 streptavidin. To further define HSC progenitors, cells were subsequently stained with FITC anti-CD34 antibodies. Stained cells were analyzed using a MoFlo cell sorter (Dako, Carpinteria, CA, USA). For identification of mast cells, a peritoneal wash was performed with 10 mL sterile phosphate-buffered saline. Collected cells were stained with anti–c-Kit (allophycocyanin) and anti–Sca-1 (Pacific Blue). Enriched HSC (Lin^−^ c-Kit^+^ Sca-1^+^) cells were sorted directly into 96% ethanol, washed extensively, and stained with propidium iodide and anti-Ki-67 (FITC) as a marker for cell cycle analysis. Whole BM erythropoietic cells were stained with CD71 (FITC) and Ter119 (phycoerythrin) antibodies.

### RNA isolation and quantitative PCR assays

For RNA isolation from tissues, a single-cell suspension was prepared using a tissue homogenizer. Cells were resuspended in TRI reagent (Sigma, St Louis, MO, USA) and RNA isolated as described by the manufacturer. First-strand complementary DNA synthesis was performed using the cDBA Synthesis Kit (Bioline, Taunton, MA, USA). Quantitative PCR was carried out using Stratagene Brilliant SYBR Green qPCR Master Mix (Agilent Technologies, Stockport, UK). Standard curves were obtained using serial dilutions of control sample. Data were normalized to β-actin. Scl messenger RNA (mRNA) primers: Scl Exon 5 F- catgttcaccaacaacaaccg Scl Exon 6 R ggtgtgaggaccatcagaaatctc; Scl primary transcript primers: Scl Exon 1 F –tatgcctgtgtgcctgtgtccttt; Scl Intron 2 R –caacactggctcccgaatacatca; β-actin primers: β-actin F –tcctggcctcactgtcca; β-actin R –gtccgcctagaagcacttgc.

### Methylcellulose colony assays

To identify progenitor colonies, single-cell suspensions of 5 × 10^4^ BM or 2 × 10^5^ spleen cells were plated in duplicate in semisolid medium (MethoCult 3434; StemCell Technologies, Vancouver, BC, Canada). Colonies were counted and identified after 7 to 10 days in culture. To detect CFU-megakaryocyte, cells were plated in duplicate in collagen-based medium (MegaCult-C; StemCell Technologies). After 6 to 8 days in culture, slides were dehydrated, fixed, and stained with acetylthiocholiniodide (Sigma). Cultures were performed according to the manufacturer's protocol.

### Phenylhydrazine treatment

Anemia was induced with phenylhydrazine (Sigma) injected intraperitoneally (60 mg/kg body weight) at day 1 and day 2. At day 4, mice were analyzed.

### Bone marrow transplantations

Young adult recipient mice Ly5.1 (C57/black) underwent whole body γ-irradiation with 12 Gy to ablate their BM. This was followed immediately by tail vein injection of 1 × 10^6^ (Ly5.2) cells in a ratio of 1:1 recipient to donor WT or *Scl*^*Δ19/Δ19*^ whole BM cells. Animals were bled 4 and 12 weeks after BM transplantation. All animal procedures were carried out under British Home Office procedural and ethical guidelines.

### Chromatin immunoprecipitation assay

A single-cell suspension from fetal liver isolated from E14.5 embryos was cross linked with 0.4% formaldehyde and nuclear extracts were prepared. Nuclear extracts were sonicated to shear the DNA and precleared with rabbit IgG (Sigma) and Protein G agarose beads (Roche, Roche Applied Science, Burgess Hill, UK). Specific antibodies for H3K9me3, H3K9me2, H3K4me3, and H3K9Ac (Upstate Biotechnology, Inc., Lake Placid, NY, USA) were added at 2.5 μg per 1 × 10^7^ lysed cells and incubated overnight at 4°C. Immunoprecipitated DNA material was released by reverse cross linking and enriched DNA fragments were purified and used for amplification by qPCR. The primers used for the regional analysis are as described [14].

### Statistical analysis

The means of each dataset were analyzed using Student's *t* test with a two-tailed distribution and assuming equal sample variance.

## Results

### Scl^Δ19/Δ19^ are viable and have normal mature Scl expression in hematopoietic lineages

**Figure 1:**
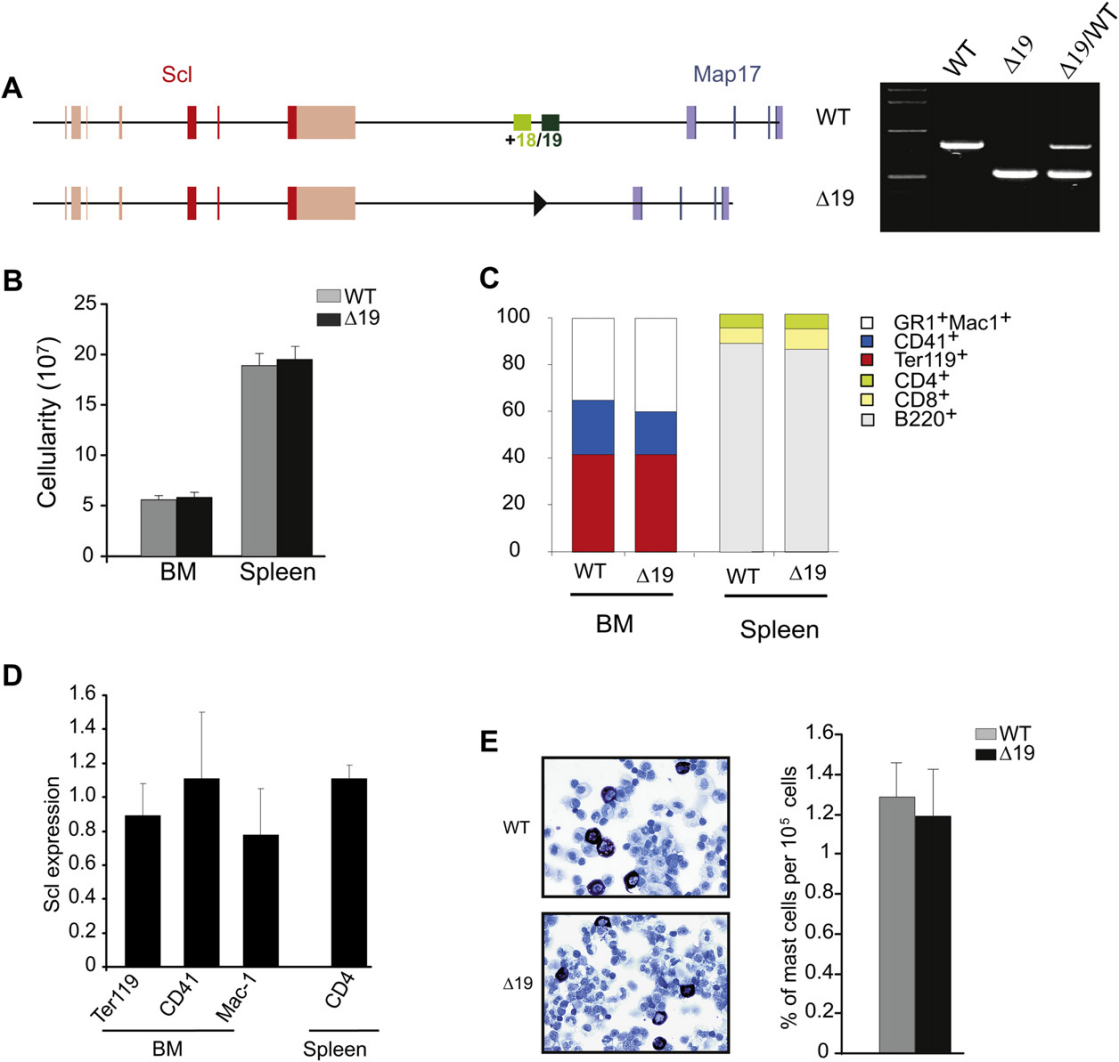

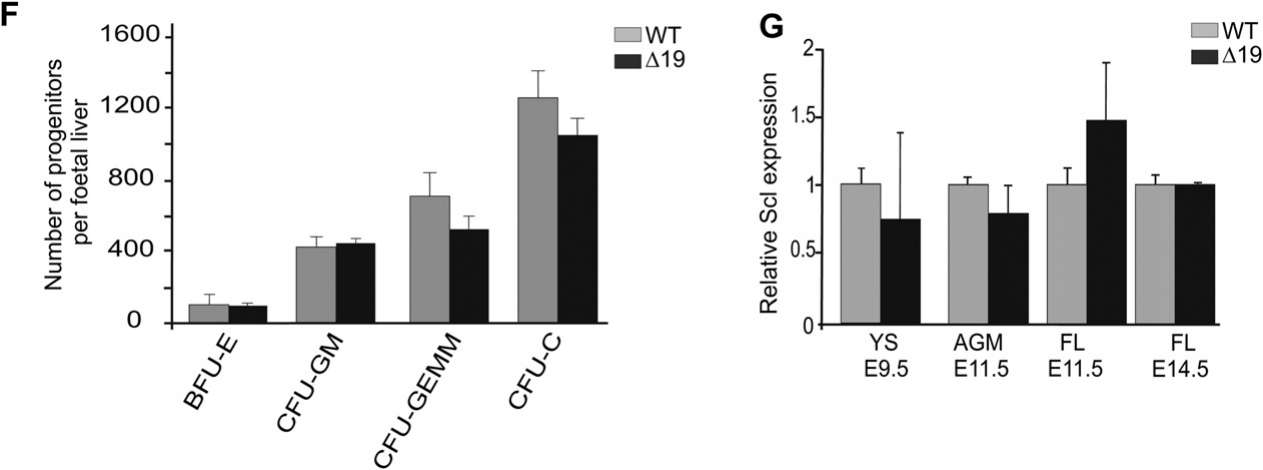
Scl^*Δ19/Δ19*^ mice are viable and have normal mature hematopoietic lineages. (**A**) Left panel shows schematic representation of the *Scl* alleles used in this study: Scl^WT/WT^ and Scl^*Δ19/Δ19*^ locus with the deletion of a 2.4-kb region containing both the +18 (light green bar) and +19 enhancers (dark green bar). Black triangle represents loxP site remaining in the genome after Cre recombination. Scl exons are depicted in red and the Map17 exons in blue. Right panel shows PCR genotyping analysis of WT (Scl^WT/WT^), homozygous (Scl^*Δ19/Δ19*^), and heterozygous (Scl^*Δ19/WT*^) knockout alleles. In the first lane is 1-kb DNA marker. (**B**) Analysis of total cellularity from the BM and spleen in Scl^WT/WT^ and Scl^*Δ19/Δ19*^ adult mice. (**C**) Percentage of granulocytes (Gr1^+^Mac1^+^), megakaryocytes (CD41^+^), and erythrocytes (Ter119^+^) cells in BM and T cells (CD4^+^, CD8^+^) and B cells (B220^+^) in spleen of Scl^WT/WT^ and Scl^*Δ19/Δ19*^ mice. (**D**) Scl expression in mature blood lineages of the BM and spleen in Scl^*Δ19/Δ19*^ mice. Data are presented as relative expression to Scl^WT/WT^. Erythroid cells were sorted using Ter119 antibody, megakaryocytes using CD41, macrophages using Mac-1 and T cells from spleen using CD4. (**E**) Mast cells are normal in Scl^*Δ19/Δ19*^ mice. Left panel shows peritoneal cells stained with Toludine blue and Metachromatic staining of mast cells. Right panel shows quantitative analysis of mast cells (cKit^+^ Sca1^+^) from peritoneal wash in Scl^WT/WT^ and Scl^*Δ19/Δ19*^ mice.

We have previously reported that chimeric mice created from Scl^*Δ19/Δ19*^ ES cells, where a 2.5-kb fragment containing the Scl +18 and Scl +19 elements was deleted, show contribution of the deleted cells in all hematopoietic lineages [16]. However, in these studies, there was still WT cell-derived hematopoiesis that might have masked quantitative effects of the deletion. Therefore, to study the effects of the deletion on hematopoiesis from embryo to adult, we generated *Scl*^*Δ19/Δ19*^ homozygous knockout mice by crossing *Scl*^*Δ19/WT*^ heterozygous mice (Fig. 1A).

**Supplementary Figure E1:**
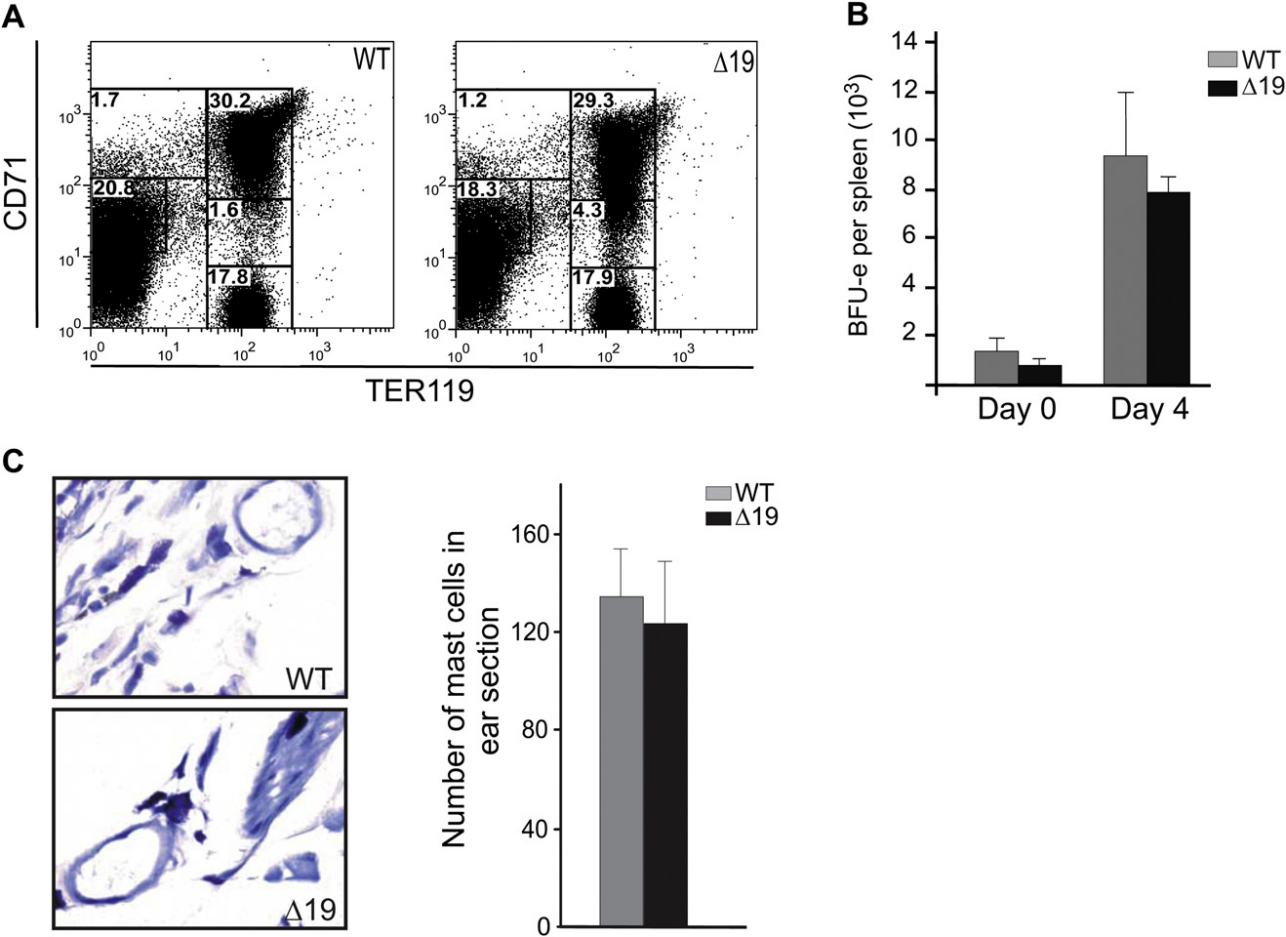
Scl^*Δ19/Δ19*^ mice hematopoietic phenotype. (**A**) Representative fluorescence-activated cell sorting analysis of CD71 and Ter119 staining of adult BM shows a normal erythroid differentiation profile for WT and Scl^*Δ19/Δ19*^ mice. (**B**) Erythropoiesis of 6- to 8-week-old sex- and age-matched Scl^WT/WT^ and Scl^*Δ19/Δ19*^ mice was stressed with phenylhydrazine (PHZ). Burst-forming erythriod (BFU-e) colonies were increased equally in the spleens of Scl^WT/WT^ and Scl^*Δ19/Δ19*^ mice 4 days after the first injection. (**C**) Left panel shows representative ear sections of Scl^WT/WT^ and Scl^*Δ19/Δ19*^ stained with Toluidine blue and quantification of mast cells is summarized in histogram.

*Scl*^*Δ19/Δ19*^ and WT mice were born at Mendelian ratios from heterozygous crosses, demonstrating that the deletion of the enhancer does not result in embryonic lethality. Hematological parameters in the peripheral blood of WT and *Scl*^*Δ19/Δ19*^ mice were comparable at both 6 to 12 weeks and 78 to 86 weeks of age (Table 1). Adult BM and spleen cellularity of *Scl*^*Δ19/Δ19*^ and WT mice were also comparable (Fig. 1B). Surface marker (Ter119, Mac-1 and Gr-1, CD41, B220, CD4, and CD8) analysis in adult BM and spleen did not reveal any significant differences between WT and *Scl*^*Δ19/Δ19*^ mice (Fig. 1C). More detailed analysis of the erythroid lineage using the CD71 and Ter119 markers failed to reveal any abnormalities in erythropoiesis in adult BM and spleen (Supplementary Figure E1A; online only, available at www.exphem.org). In addition, phenylhydrazine treatment of *Scl*^*Δ19/Δ19*^ mice induced a normal stress erythropoiesis reaction (Supplementary Figure E1B; online only, available at www.exphem.org).

Real-time semi-quantitative PCR performed with primers specific for the *Scl* gene [16] showed that expression levels of *Scl* in erythroid (Ter119^+^), myeloid (Mac-1^+^), megakaryocytic (CD41^+^), and T-cell (CD4^+^) lineages were similar for WT and *Scl*^*Δ19/Δ19*^ mice (Fig. 1D). It has recently been shown that Scl plays a role in mast cells [21]. However, *Scl*^*Δ19/Δ19*^ peritoneal and connective tissue mast cells were morphologically, phenotypically, and quantitatively normal (Fig. 1E and Supplementary Figure E1C; online only, available at www.exphem.org). To determine whether deletion of the Scl +19 enhancer affects embryonic/fetal hematopoiesis, we quantified the number of progenitors in the fetal liver using methylcellulose-based colony assay. No difference was detected in burst-forming unit erythroid, CFU–granulocyte-macrophage (GM), CFU- multipotential progenitor cells, and CFU in culture from WT and *Scl*^*Δ19/Δ19*^ fetuses (Fig. 1F). The expression level of Scl was unaltered in yolk sac at E9.5, aortagonad-mesonephros at E11.5, and fetal liver at E11.5 and E14 (Fig. 1G).

These results indicate that deletion of the Scl +19 enhancer results in viable mice with a normal distribution and levels of Scl expression in embryonic and adult tissues and no effect on BM cellularity or fetal progenitor numbers.

### Scl +19 enhancer deletion increases HSC number but does not alter the differentiation capacity of progenitor cells

**Figure 2:**
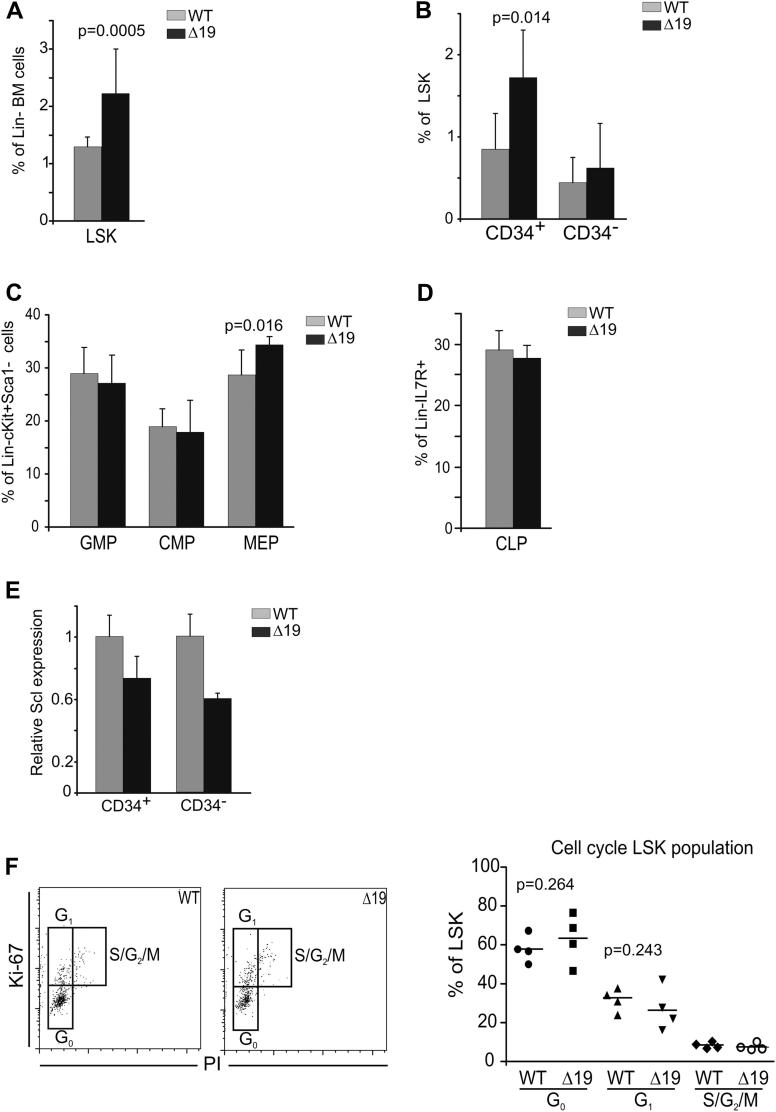
Phenotypic analysis of progenitor cells in adult BM of WT and Scl^*Δ19/Δ19*^ mice. (**A**) *Scl*^*Δ19/Δ19*^ animals have an increased number of Lin^−^Sca1^+^Kit^+^ cells compared with the Scl^WT/WT^. Histogram shows percentage of Sca1^+^Kit^+^ cells within the Lin^−^ BM cells of Scl^WT/WT^ and Scl^*Δ19/Δ19*^ mice. (**B**) No significant difference is observed in the number of long-term CD34^−^ stem cells in the two genotypes; however, there is a 2-fold increase in short-term CD34^+^ stem cells in Scl^*Δ19/Δ19*^ mice. Histogram show percentage of CD34^+^ and CD34^−^ within the LSK population in BM. (**C**) Analysis of BM progenitor population reveals a normal population of granulocyte-macrophage progenitors (GMPs) and common myeloid progenitors but an increase in the megakaryocytic erythroid progenitor (MEP) population in Scl^*Δ19/Δ19*^. (**D**) No difference was observed in the number of common lymphoid progenitors (CLPs) between WT and Scl^*Δ19/Δ19*^ animals. (**E**) Expression level of *Scl* in short-term (LSK CD34^+^) and long-term (LSK CD34^−^) stem cells. (**F**) Cell cycle fluorescence-activated cell sorting analysis on sorted LSK population stained for the Ki-67 and propidium iodide (PI) in Scl^WT/WT^ and Scl^*Δ19/Δ19*^ mice. Left panel shows representative plots and right panel shows quantification results. Analysis was performed on six animals from each genotype.

As the Scl +19 enhancer has been shown to be mainly active in progenitor cells [17], we next performed a more detailed analysis of hematopoietic progenitors. The data obtained are shown in Figure 2 as a percentage of the specific progenitor population in lineage-depleted BM. The most significant increase in cell number (1.5-fold; *p* < 0.005) was found in the lineage-negative Sca1^+^ cKit^+^ (LSK) population (Fig. 2A). A similar phenotype was observed upon Scl deletion in adult mice [12]. Further analysis of the LSK population using the CD34 marker revealed a significant increase in the LSK CD34^+^ population, whereas no difference was observed in the LSK CD34^−^ population (Fig. 2B). The LSK CD34^+^ population is enriched for ST-HSCs, while the LSK CD34^−^ population is enriched for the more immature LT-HSCs [22].

A smaller but statistically significant increase of 20% was also observed in megakaryocytic erythroid progenitors, but no significant difference was observed in granulocyte-macrophage progenitors, common myeloid progenitors (Fig. 2C), or common lymphoid progenitors (Fig. 2D). To complement the data, in vitro colony assays were performed with adult BM and spleen to assess the differentiation potential of *Scl*^*Δ19/Δ19*^ cells (Table 2). CFU–multipotential progenitor cells, CFU-GM, burst-forming unit erythroid, and CFU-megakaryocyte colony numbers were unaffected in the adult BM. By contrast, we observed a twofold increase in CFU-GM in the spleen of *Scl* knockout mice, without any sign of spleen enlargement.

The increased number of LSK cells in *Scl*^*Δ19/Δ19*^ mice suggested that deletion of the +19 enhancer might play an important role in immature HSCs. We purified CD34-positive and negative fractions from the LSK population and analyzed *Scl* expression (Fig. 2E). The level of *Scl* mRNA in the LSK CD34^−^ population was almost half that of the WT mice (*p* = 1.7 × 10^−6^), whereas that in LSK CD34^+^ population was reduced by only 20% (*p* = 0.0087). To test the possibility that the increased number of cells within the LSK population is due to cell cycle arrest, we stained the purified LSK population for the cell cycle marker Ki-67 (Fig. 2F). The *Scl*^*Δ19/Δ19*^ LSK cells showed a very mild increase in G_0_ phase and a slight decrease in G_1_ phase compared with the WT mice; however, both changes fail to reach statistical significance. The S/G_2_/M phases did not differ between the two phenotypes. This showed that the +19 enhancer affects the level of Scl expression in HSCs, yet the changes in HSC numbers observed in *Scl*^*Δ19/Δ19*^ mice are not due to major alterations in cell cycle.

In summary, *Scl*^*Δ19/Δ19*^ mice exhibit an increase in short-term HSCs as assessed by flow cytometry, an increase in megakaryocytic erythroid progenitors and an increase in CFU-GM colonies in spleen. However, deletion of the Scl +19 element has no effect on the common myeloid progenitors, granulocyte-macrophage progenitors, and common lymphoid progenitor populations.

### The +19 enhancer is not required for HSC multilineage repopulation

**Figure 3:**
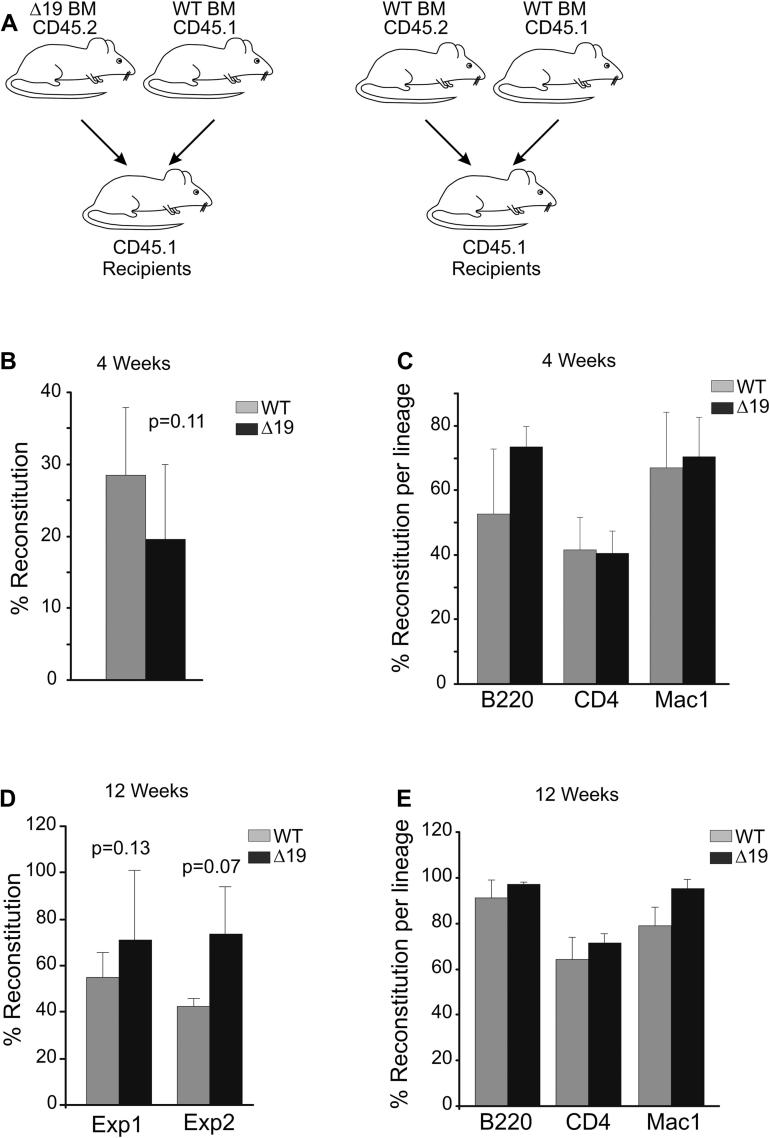
Enhanced long-term repopulation in Scl^*Δ19/Δ19*^ mice. (**A**) Outline of transplantation assay. CD45.2 Scl^WT/WT^ or Scl^*Δ19/Δ19*^ unfractionated BM was mixed with CD45.1 unfractionated BM at a ratio of 1:1. Five to six lethally irradiated CD45.1 recipients were injected with 1 × 10^6^ cells per group (Scl^*Δ19/Δ19*^ or Scl^WT/WT^ donor CD45.2). (**B**) Percentage reconstitution (CD45.2^+^ cells) measured 4 weeks post-transplantation by fluorescence-activated cell sorting (FACS) analysis of peripheral blood with CD45.1 and CD45.2 antibodies. (**C**) Recipient peripheral blood FACS analysis for different lineage markers: B cells (B220), macrophages (Mac-1), and T cells (CD4). CD45.2 donor reconstitution for each lineage is expressed as a percentage. (**D**) Percentage reconstitution measured 12 weeks post-transplantation by FACS analysis of peripheral blood with CD45.1 and CD45.2 antibodies. Two independent transplantations are shown. (**E**) Recipient peripheral blood FACS analysis for different lineage markers: B cells (B220), macrophages (Mac-1), and T cells (CD4). CD45.2 donor reconstitution for each lineage is expressed as a percentage.

We showed (Fig. 2A) that LSK numbers were increased by almost twofold in *Scl*^*Δ19/Δ19*^ mice. The ultimate assay for HSC function is the long-term competitive repopulation assay. A long-term competitive repopulation assay was performed to assess the effect of the deletion on HSC function, using the CD45.1–CD45.2 system to measure blood chimerism in transplant recipients. Donor WT or *Scl*^*Δ19/Δ19*^ cells were CD45.2, whereas competitor cells and recipient mice were CD45.1 (Fig. 3A). For engraftment studies we used a 1:1 ratio of donor to competitor and a total of 1 × 10^6^ unfractionated donor and competitor BM cells were injected per recipient. Results are shown in Figure 3 as a percentage of chimerism 4 and 12 weeks post-transplantation.

Short-term engraftment was analyzed at 4 weeks post-transplantation. Two independent transplantations were performed and the results are shown in Figure 3B and C. Short-term engraftment was found to be somewhat compromised for *Scl*^*Δ19/Δ19*^ cells, which is in agreement with previous phenotypic observations in *Scl* conditional knockout animals [12]. The observed reduction in engraftment by the *Scl*^*Δ19/Δ19*^ is consistent between experiments, but fails to reach statistical significance.

Long-term engraftment was analyzed, at 12 weeks post-transplantation. Although no significant defect was observed with the *Scl*^*Δ19/Δ19*^ donor cells, there was a bias for the *Scl*^*Δ19/Δ19*^ cells to engraft better than the WT cells (Fig. 3D). However, when the percentage of donors for each lineage was calculated (Fig. 3E), we did not observe any significant differences for any lineage engraftment contribution.

### Redundancy of the Scl regulatory elements

**Figure 4:**
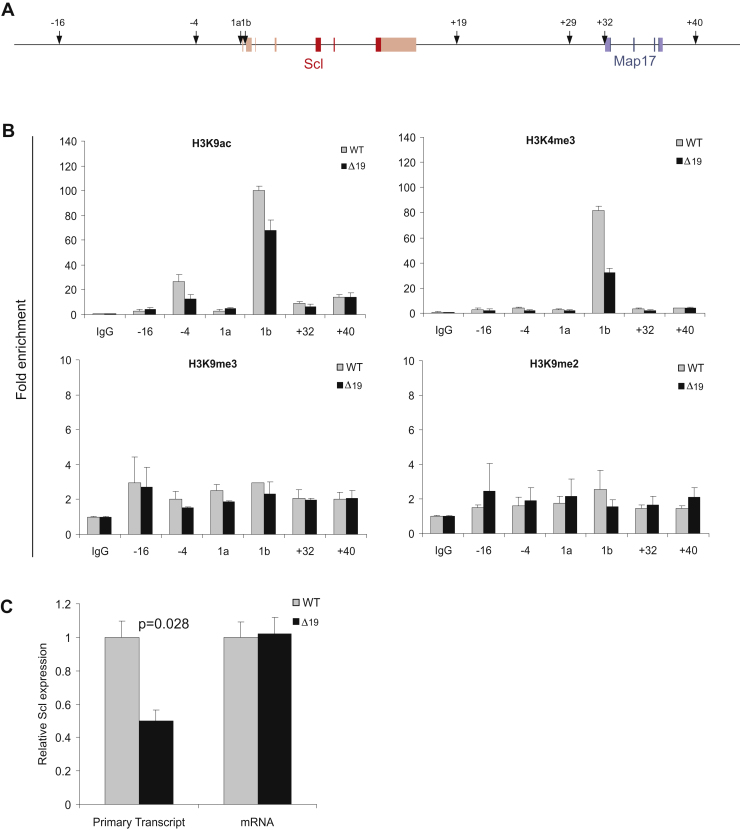
Decreased activity at Scl promoters in Scl^*Δ19/Δ19*^ mice. (**A**) The mouse *Scl* and neighboring *Map17* gene loci. Scl exons are depicted in red and the Map17 exons in blue. Arrows indicate functionally characterized Scl regulatory elements. The number above each arrow indicates the position, in kb, of the enhancer in relation to the Scl promoter 1a. 1a- Scl Promoter 1a; 1b- Scl Promoter 1b. (**B**) ChIP assay for active (H3K4me3 and H3K9Ac) and repressive (H3K9me3 and H3K9me2) chromatin marks. Fold enrichment was compared with that of the IgG control. (**C**) qRT-PCR analysis of Scl primary transcript and mRNA expression in E14.5 fetal liver cells shows a reduction of primary transcript levels in enhancer deleted cells.

We previously identified several different regulatory elements that control the spatial and temporal expression of the *Scl* gene in vitro and in vivo [16,17,23], which are summarized in Figure 4A. The Scl +19 element is controlled by a multiprotein complex that includes GATA-2, Fli-1, and Elf-1 [16]. To identify possible redundancy between the +19 enhancer and other Scl regulatory elements, we performed chromatin immunoprecipitation (ChIP) assays on E14.5 fetal liver cells with markers for repressive (H3K9me3 and H3K9me2) and active (H3K4me3 and H3K9Ac) chromatin. Using the two markers for repressive chromatin, we did not observe any differences between the Scl^*Δ19/Δ19*^ and Scl^WT/WT^ fetal liver cells at different elements of the *Scl* locus (Fig. 4B, bottom panels). By contrast, ChIP performed with active euchromatin marks revealed changes at Scl promoter region 1b (Fig. 4B, top panels). Interestingly, both active euchromatin marks are reduced in *Scl*^*Δ19/Δ19*^ fetal liver cells, indicating that the Scl +19 element is required for enhanced activity of the Scl promoter. Another region that was significantly decreased by the Scl +19 deletion was the Scl-4 enhancer located 4 kb upstream of the start site of the Scl coding sequence. Chromatin analysis of the endogenous gene locus therefore demonstrates direct functional consequences of the deletion of a distal enhancer on the status of the Scl promoter region.

Surprisingly, the expression of Scl mRNA is unaltered in Scl^*Δ19/Δ19*^ E14.5 fetal livers (Fig. 1G and Fig. 4C), suggesting post-transcriptional compensation. To test this hypothesis, we quantified the amount of Scl primary transcript present in WT and mutant fetal liver cells from the same litters by qPCR using primers spanning the exon 1b:intron1 boundary (Fig. 4C). This analysis demonstrated a 50% reduction of primary transcript in mutant cells, consistent with the ChIP data over the Scl promoter 1B. Taken together therefore, our results reveal post-transcriptional compensatory mechanisms as the likely cause for the similar levels of steady-state mRNA in wild type and enhancer-deleted cells.

## Discussion

The murine Scl locus has emerged, over the past decade or so, as a paradigm gene locus for studying transcriptional control mechanisms in blood stem and progenitor cells [14–18,20,23–25]. Concerted biochemical, comparative genomics, and transgenic studies led to the identification of three distinct regions (−4, +19, and +40) active in progenitor cells, with the +19 enhancer being the most specific HSC enhancer element [14,16,17,19,23]. Importantly, none of the previous studies of Scl regulation had performed extensive analysis of steady-state hematopoiesis after deletion of regulatory elements from the endogenous locus. Here we report a comprehensive phenotypic analysis of mice lacking the Scl +19 enhancer. Scl^*Δ19/Δ19*^ are viable with specific defects in the hematopoietic stem/progenitor cell compartment. However, Scl^*Δ19/Δ19*^ HSCs did not display any significant imbalance in the production of mature cells, and were able to function in transplantation assay. Rather than being accompanied by compensatory increases in active chromatin marks elsewhere in the locus, deletion of the +19 enhancer causes a reduction of active histone marks at the −4 enhancer and Scl promoters.

The observation of relatively mild phenotypes after the deletion of regulatory elements is a common finding. Early studies on β-globin enhancers showed only moderate reductions in expression [26–28] and similar results were also obtained after the deletion of the α-globin upstream enhancer [29]. Perhaps the most publicized failure to elicit strong phenotypes after deletion of enhancer elements comes with the demonstration that germline deletion of four ultra-conserved elements did not cause any major phenotype [30]. Given that the complete knockout of genes such as Scl or globins results in major phenotypes, the minor phenotypes after enhancer knockouts have commonly been attributed to compensation by additional regulatory elements with overlapping activity, within the same gene locus. Most recently, Snow and colleagues [31] have shown that deletion of a Gata2 enhancer, predicted to be responsible for the Gata2-dependent positive feedback loop in HSCs, had no major phenotypical consequences despite a significant reduction in Gata2 expression and the lack of phenotype was again attributed to redundancy with another cis-regulatory element.

We have previously demonstrated that the Scl +19 element is controlled by a multiprotein complex that includes GATA-2, Fli-1, and Elf-1 [16]. Using whole-genome transcription factor binding mapping by ChIP-Seq, we have recently identified other important hematopoietic regulators that also bind to the Scl +19 enhancer, including the Ets factors Pu.1 and Erg [32,33]. Like GATA-2 and Fli1, Pu.1 and Erg were also bound to other Scl enhancers (−4 and/or +40 regions), suggesting that the entire Scl transcriptional domain serves as a target for these upstream regulators. Of note, the recurrent presence of overlapping sets of transcription factors at several enhancers of a particular gene locus may be one of the reasons that deletion of individual enhancer elements often causes only mild phenotypes.

HSCs have the ability of self-renewal, extensive proliferation, and to contribute to all lineages of hematopoiesis. Adult HSCs can be divided into LT-HSC and ST-HSC populations. We observed a significant increase in the HSC-enriched LSK population in *Scl*^*Δ19/Δ19*^ mice. Of note, a similar phenotype has been described previously using conditional deletion of *Scl* in adult HSCs [12], demonstrating the specific function of the +19 element in HSC expression of Scl. Furthermore, even though the ST-HSCs and LT-HSCs in our *Scl*^*Δ19/Δ19*^ mouse model showed a reduced level of Scl expression, there was no major defect in the long-term competitive repopulation assay, which again is consistent with analysis of adult HSC function in conditional Scl knockout mice [10–12]. However, unlike the conditional Scl knockout, the Scl^*Δ19/Δ19*^ mutation is constitutive and, therefore, present at the early developmental stages where Scl function is absolutely critical [6–8]. Importantly, the data presented here suggests that the main compensatory mechanism for loss of the Scl +19 enhancer may not be through alternative regulatory elements. This notion is based on the observation that, despite the lack of change in steady-state mRNA levels, active histone marks over the Scl promoter as well as the levels of Scl primary transcripts were reduced significantly after deletion of the +19 enhancer from the endogenous Scl locus. It has been shown previously that Scl mRNA stability is enhanced during terminal erythroid maturation [34], thus providing a potential mechanism for post-transcriptional control of Scl mRNA steady-state levels.

It is, of course, not unexpected that the activity of important regulators such as Scl is controlled at multiple levels from transcription [14–19,23,24,35] to post-transcriptional [34,36] to translational [37] to post-translational [38] control. Compensation for loss of a transcriptional regulatory element can occur at any of those levels. The studies reported here have therefore not only demonstrated a remarkable tissue-specific function for the Scl +19 stem cell enhancer, but also highlight the need to consider nontranscriptional compensatory mechanisms when interpreting enhancer knockout phenotypes.

## Figures and Tables

**Table 1:** Hematological parameters of *Scl*^*Δ19/Δ19*^ and WT mice

Age (wks)	Genotype	N	RBC (10^3^/μL)	Hgb (g/dL)	Hct (%)	Plt (10^3^/μL)	WBC (10^3^/μL)	Lympho (%)	Mono (%)	Gran (%)
6–12	WT	26	9.5 ± 1.3	17.2 ± 0.7	53.5 ± 4.0	1107 ± 234	7.7 ± 2.0	78.7 ± 5.3	4.2 ± 0.5	17.1 ± 5.1
	Δ19	26	9.6 ± 1.2	16.8 ± 0.8	53.1 ± 3.3	965 ± 209	7.4 ± 2.2	78.7 ± 5.7	4.3 ± 1.1	17 ± 4.9
78–86	WT	12	10.1 ± 1.5	14.2 ± 2.4	46.0 ± 9.1	1551 ± 254	11.3 ± 7.2	64 ± 13.4	6.8 ± 2.4	29.2 ± 11.5
	Δ19	13	10.6 ± 1.3	14.5 ± 2.1	48.3 ± 8.2	1527 ± 353	13.7 ± 5.9	64.3 ± 12.1	6.8 ± 1.8	29 ± 10.7

Gran = granulocytes; Hct = hematocrit; Hgb = hemoglobin; Lympho = lymphocytes; mono = monocytes; Plt = platelets; RBC = red blood cells; WBC = white blood cells. Peripheral blood parameters were measured from age- and sex-matched young (6–12 weeks) and old (78–86 weeks) mice.

**Table 2:** Hematopoietic progenitors are normal in *Scl*^Δ19*/*Δ19^ mice

Tissue	Genotype	BFU-e	CFU-GM	CFU-GEMM	CFU-MK
BM	WT	2 ± 1	28 ± 16	4 ± 2	13 ± 4
	Δ19	2 ± 1	26 ± 12	3 ± 3	15 ± 1
Spleen	WT	3 ± 1	17 ± 2	5 ± 2	9 ± 6
	Δ19	4 ± 1	34 ± 6	5 ± 2	11 ± 9

Methylcellulose-based colony assays where performed in whole BM and spleen from WT and Δ19 mice. The numbers shown are per 5 × 10^4^ BM and 2 × 10^5^ spleen cells for burst-forming units-erythroid (BFU-e), colony-forming units–granulocyte macrophage (CFU-GM), CFU–multipotential progenitors (CFU-GEMM), and CFU- megakaryocytes (CFU-MK). Results represent six age- and sex-matched mice of each genotype. Values are expressed as mean ± standard deviation.
